# Clinical and immunovirological status in children, adolescents and young adults with early-life-acquired HIV: a Spanish multi-cohort analysis since 2020

**DOI:** 10.1093/jac/dkag079

**Published:** 2026-03-11

**Authors:** Laura Tarancon-Diez, Beatriz Lazaro-Martin, Javier Goicoechea-Martínez, Raquel Domínguez-Romero, Jorge Gómez Sirvent, Elisa Garrote, Laura Minguell Domingo, Eloísa Cervantes-Hernández, César Gavilán, Clàudia Fortuny, Jose Ignacio Bernardino, Cristina Diez, Marta Montero-Alonso, Cristina Roca-Oporto, Antonio Ocampo, Ana Belén Jiménez, Neus Rius, Nuria López Segura, Luis Escosa-García, Luis Prieto, Jose Tomás Ramos, Maria Luisa Navarro-Gomez

**Affiliations:** Department of Pediatrics, Pediatric Infectious Diseases Unit, Gregorio Marañón University Hospital, Gregorio Marañón Research Health Institute (IiSGM), Madrid, Spain; Biomedical Research Centre Network for Infectious Diseases (CIBERINFEC), Carlos III Health Institute, Madrid, Spain; Department of Pediatrics, Pediatric Infectious Diseases Unit, Gregorio Marañón University Hospital, Gregorio Marañón Research Health Institute (IiSGM), Madrid, Spain; Biomedical Research Centre Network for Infectious Diseases (CIBERINFEC), Carlos III Health Institute, Madrid, Spain; Department of Pediatrics, Pediatric Infectious Diseases Unit, Gregorio Marañón University Hospital, Gregorio Marañón Research Health Institute (IiSGM), Madrid, Spain; Department of Pediatrics, Pediatric Infectious Diseases Unit, Gregorio Marañón University Hospital, Gregorio Marañón Research Health Institute (IiSGM), Madrid, Spain; Department of Pediatrics, University Hospital Nuestra Señora de la Candelaria, Santa Cruz de Tenerife, Spain; Department of Pediatrics, Hospital Universitario Basurto, Bilbao, Vizcaya, Spain; Department of Pediatrics, Hospital Universitari Arnau de Vilanova, Lleida, Spain; Department of Pediatrics, Hospital Universitario Virgen de la Arrixaca, Murcia, Spain; Department of Pediatrics, Hospital San Juan de Alicante, Sant Joan d'Alacant, Spain; Pediatric Infectious Diseases and Microbiome Research Group, Institut de Recerca Sant Joan de Déu, Spain; Departament de Cirurgia I Especialitats Medicoquirúrgiques, Facultat de Medicina I Ciències de la Salut, Universitat de Barcelona, Barcelona, Spain; Biomedical Research Centre Network for Epidemiology and Public Health (CIBERESP), Carlos III Health Institute, Madrid, Spain; Biomedical Research Centre Network for Infectious Diseases (CIBERINFEC), Carlos III Health Institute, Madrid, Spain; HIV and Infectious Diseases Unit, Hospital Universitario La Paz, Instituto de Investigación Hospital Universitario La Paz (IdiPAZ), Madrid, Spain; Biomedical Research Centre Network for Infectious Diseases (CIBERINFEC), Carlos III Health Institute, Madrid, Spain; HIV and Infectious Diseases Unit, Hospital General Universitario Gregorio Marañón, Instituto de Investigación Sanitaria Gregorio Marañón (IiSGM), Madrid, Spain; Infectious Diseases Unit, La Fe University and Polytechnic Hospital, Valencia, Spain; Institute of Biomedicine of Seville (IBiS), Virgen del Rocio University Hospital, Spanish National Research Council (CSIC), University of Seville, Clinical Unit of Infectious Diseases, Microbiology and Parasitology, Seville, Spain; Complejo Hospitalario Universitario de Vigo, Vigo, Spain; Department of Pediatrics, Hospital Universitario Fundación Jiménez Díaz, Madrid, Spain; Department of Pediatrics, Hospital Universitari San Joan de Reus, Tarragona, Spain; Department of Pediatrics, Hospital del Mar, Barcelona, Spain; Biomedical Research Centre Network for Infectious Diseases (CIBERINFEC), Carlos III Health Institute, Madrid, Spain; Department of Pediatrics, Infectious and Tropical Diseases, La Paz University Hospital, Hospital La Paz Institute for Health Research (Idipaz), Madrid, Spain; Biomedical Research Centre Network for Infectious Diseases (CIBERINFEC), Carlos III Health Institute, Madrid, Spain; Department of Paediatrics, Pediatric Research and Clinical Trials Unit (UPIC), Fundación Para la Investigación Biomédica del Hospital 12 de Octubre, Instituto de Investigación Sanitaria Hospital 12 de Octubre (I+12), Madrid, Spain; Universidad Complutense de Madrid, Madrid, Spain; Biomedical Research Centre Network for Infectious Diseases (CIBERINFEC), Carlos III Health Institute, Madrid, Spain; Department of Paediatrics, Pediatric Research and Clinical Trials Unit (UPIC), Fundación Para la Investigación Biomédica del Hospital 12 de Octubre, Instituto de Investigación Sanitaria Hospital 12 de Octubre (I+12), Madrid, Spain; Universidad Complutense de Madrid, Madrid, Spain; Department of Pediatrics, Pediatric Infectious Diseases Unit, Gregorio Marañón University Hospital, Gregorio Marañón Research Health Institute (IiSGM), Madrid, Spain; Biomedical Research Centre Network for Infectious Diseases (CIBERINFEC), Carlos III Health Institute, Madrid, Spain; Universidad Complutense de Madrid, Madrid, Spain

## Abstract

**Objectives:**

ART has significantly improved survival among children, adolescents and young adults who acquired HIV perinatally or during early childhood (early-life acquired HIV, ELHIV). However, challenges persist, including virological failure (VF) and suboptimal immune recovery. This study aimed to describe clinical, virological and immunological outcomes of ELHIV individuals in Spain since 2020, and to identify factors associated with VF and impaired immune recovery.

**Methods:**

A multicentre, retrospective cohort study was conducted using data from 642 ELHIV individuals actively followed in the CoRISpe and CoRISpe-FARO cohorts. Data included demographics, ART history, virological suppression (viral load ≤50 copies/mL), CD4/CD8 ratio and CDC immunological categories. Logistic regression identified factors influencing VF and immune progression.

**Results:**

The median age of participants was 24 years, with 67.6% aged ≥18. Most (93.6%) acquired HIV via vertical transmission, with ART initiated at a median age of 1.93 years. At the time of analysis, 99.1% were on ART. Although 81.1% achieved virological suppression, 10.5% experienced VF, associated with PI-based regimens, independent of age, and a lower CD4 nadir. Immune recovery, defined as a CD4/CD8 ratio ≥1, was achieved by 52.3%. Impaired recovery was linked to older age at ART initiation and lower CD4 nadir, particularly among adolescents (12–18 years) and young adults. Children (<12 years) showed better immune profiles, with 97.8% achieving CD4 counts ≥500 cells/mm³.

**Conclusions:**

Early ART initiation and tailored interventions are essential to optimize outcomes in ELHIV populations. PI-based regimens were a risk factor for VF, whereas integrase strand transfer inhibitors appeared protective. Adolescents and young adults require targeted support to improve adherence and immune recovery, aligning with UNAIDS goals.

## Introduction

Antiretroviral treatment (ART) has dramatically decreased mortality among individuals who acquired HIV perinatally or during childhood (early-life acquired HIV, ELHIV) in high-income countries.^1,2^ This has transformed HIV into a chronic disease,^3^ with children reaching and significantly surpassing adulthood.^4^ Maintaining long-term viral suppression is crucial for all HIV patients, but supporting ELHIV presents particular challenges. ELHIV individuals face lifelong exposure to the deleterious effects of the virus and its treatments, often involving older and more toxic drugs initiated during physiological immaturity, with limited efficacy and poor adherence to ART during childhood and adolescence. Those who reach adulthood have often been exposed to suboptimal ART regimens, including nucleoside analogues in mono- or dual therapy, non-nucleoside reverse-transcriptase inhibitors (NNRTIs) and first-generation protease inhibitors (PIs).^5^ Several studies of paediatric cohorts have reported high virological failure (VF) rates, particularly during adolescence, compared with those who acquired HIV in adulthood.^6–9^ Furthermore, a European study reported a cumulative incidence estimate for mortality at the age of 15 of 0.8%.^10^ These concerning data highlight the need to prioritize adolescents in optimizing management strategies.

Data on immunological recovery among ELHIV individuals are limited. Concerns exist regarding their long-term immunological status due to poorer virological control, although greater thymic activity in children^11^ might offer greater potential for immune recovery.^12^ However, other studies suggest that thymic function recovery is independent of age and related to peripheral CD4 cell depletion and HIV suppression.^13^ In ELHIV individuals, immune recovery has been associated with younger age, higher CD4 T cell count and CD4 nadir at ART initiation, whereas severe immunosuppression is linked to impaired CD4 recovery.^5,14–17^ The CD4/CD8 ratio is considered a good predictor of long-term immune recovery.^18^ Despite long-term viral suppression, an inverted CD4/CD8 ratio, or a value <1, is associated with a higher risk of morbidity in adults^19^ and with T cell activation, senescence and inflammation not only in HIV adults, but also in ELHIV individuals.^20,21^ Ultimately, these conditions can lead to the early development of non-AIDS events.^22–24^

Despite advances, there are still few studies describing the current immunovirological outcomes among ELHIV individuals in high-income countries, many of whom have now reached adulthood. In this context, most available data on ELHIV individuals come from paediatric-only populations or cohorts with limited follow-up across the transition to adult care. Moreover, few studies have specifically evaluated outcomes beyond 2020, a milestone year for the assessment of global HIV strategies. Spain provides a particularly informative setting, with universal access to ART, long-standing national paediatric HIV surveillance, and structured transition from paediatric to adult HIV units. The integration of data from the CoRISpe and CoRISpe-FARO cohorts allows for a comprehensive evaluation of children, adolescents and young adults with ELHIV within a single national healthcare system.

Therefore, this study aimed to describe the clinical, virological and immunological status of children, adolescents and young adults with ELHIV in Spain since 2020, and to identify age-specific patterns and factors associated with VF and immune recovery. The present study focused on the period since 2020, a key milestone set by UNAIDS to evaluate progress toward the 90-90-90 global targets (90% diagnosed, 90% on ART, and 90% virally suppressed).^25^ This timeframe allows us to assess whether ELHIV individuals have benefited from these efforts and to identify persistent challenges in this population. Additionally, we sought to identify factors associated with VF and immune progression to determine possible risk factors that could impact the clinical progression of this population.

## Materials and methods

### Study design and participants

A multicentre, observational and retrospective cohort study was performed on people living with HIV with active follow-up since 2020 within the Spanish cohort of HIV children and adolescents (CoRISpe), and patients with HIV acquired perinatally or during childhood transferred to adult units (CoRISpe-FARO).^4,26^ Analysis was designed as a cross-sectional evaluation of immunovirological status during the period 2020–2023, anchored to the UNAIDS 2020 milestone. Clinical and virological data were collected from the first medical record until December 2023. The collected data included: origin, sex, date of birth, HIV transmission route, HIV diagnosis date, ART experience, nadir CD4, CD4 and CD8 count (percentage and cells/mm^3^) from last clinical visit, HIV viral load (VL) (HIV-1 RNA copies/mL of plasma) from the last clinical visit, HCV coinfection, HBV coinfection, and biochemical and haematological data from the last clinical visit.

The study was approved by the ethics committee of Hospital General Universitario Gregorio Marañón (HGUGH) in Madrid, Spain (acta08/2024) and by the CoRISpe and CoRISpe-FARO scientific committees. Written informed consent was obtained from parents or legal guardians of patients <12 years, and direct informed assent from patients aged ≥12 years before inclusion in CoRISpe-FARO. Data were treated with full confidentiality according to Spanish legislation.

### Definitions

Age groups were defined as children (<12 years), adolescents (12 to 18 years) and young adults (>18 years). Previous HCV and HBV infections were defined as a documented positive anti-HCV antibody with negative HCV-RNA at the last available clinical assessment (regardless of the mechanism of viral clearance), and evidence of past exposure based on serological markers (anti-HBc positivity) in the absence of active HBV infection, respectively. Virological suppression was defined as a VL ≤50 copies/mL in the last available measurement. VF was defined as at least two consecutive clinical visits with VL >50 copies/mL, where the first visit had a detectable VL and the second served as confirmation. Patients with a single detectable VL without a confirmatory second measurement were not classified as VF cases. The VL cutoff point was chosen as it was the common limit of VL detection for techniques used during the years of the study. Patients with a CD4/CD8 ratio <1 at their last clinical visit were considered to be in immune progression, whereas immune recovery was defined as a CD4/CD8 ratio ≥1 at the last clinical visit. Immunological categories were defined based on 2014 CDC criteria as: category 1 (CD4 ≥ 500 cells/mm^3^); 2 (200 ≤ CD4 < 500 cells/mm^3^); and 3 (CD4 < 200 cells/mm^3^).

### Statistics

Descriptive statistics were performed and reported in terms of absolute frequencies and percentages for qualitative data, and medians with lower and upper quartiles (IQR) for quantitative data. Factors associated with VF, CD4/CD8 ratio <1 and immunological category were assessed using logistic regression. ORs and coefficients were estimated using multivariate logistic regression models that included variables with a *P* value <0.2 in the univariate analysis. This more inclusive threshold was selected to avoid the premature exclusion of potentially relevant confounders and to ensure adequate adjustment in this observational study. Since age at ART start and age at HIV diagnosis are strongly correlated, only age at ART start was included in the multivariate model when appropriate. All reported *P* values are two-sided, and a *P* value <0.05 was considered statistically significant. The Statistical Package for the Social Sciences software (SPSS 20.0; Chicago, IL, USA) and GraphPad Prism 9.0 (GraphPad Software, Inc., San Diego, CA, USA) were used for statistical analysis and graphs.

## Results

### Study population

A total of 642 children, adolescents and young adults with ELHIV were actively followed-up since 2020 in the CoRISpe and CoRISpe-FARO cohort. General demographic, HIV-related variables and immunovirological characteristics of CoRISpe and CoRISpe-FARO patients at the last clinical visit since 2020 are shown in Table 1. In general, 46.1% were male, with a median age of 24 years (IQR: 17–30); 67.6% were older than 18 years; 76.8% were born in Spain; and 65.3% were Caucasian. Their median age at HIV diagnosis was 10 months (IQR: 3–38), and 93.6% acquired HIV by a vertical transmission route. There was only one reported death (0.15%) since 2020 in the cohort. The cause of death was attributed to complications of advanced HIV (C3), specifically disseminated *Mycobacterium celatum* infection and *Klebsiella pneumoniae* sepsis, both of which were likely exacerbated by prolonged immunosuppression (CD4 24 cells/mm³) and ART discontinuation. Regarding ART history, the median age at ART initiation was 1.93 years (IQR: 0.38–5.03), and 99.1% were on ART. The median time under ART was 22 years (IQR: 14–26), and 8.6%, 14.6% and 58% were receiving an ART regimen that included, respectively, 2 nucleoside reverse transcriptase inhibitor (NRTIs) + 1 NNRTI, 2 NRTIs + 1 PI, or 2 NRTIs + 1 integrase strand transfer inhibitor (INSTI). The ‘other ART regimens category’ included 17.6% of the subjects, who received heterogeneous strategies such as NRTI-sparing regimens, boosted dual therapies, and other non-standard combinations, often reflecting treatment simplification or historical adaptations in heavily pre-treated patients. Due to their heterogeneity and limited sample size of individual regimens, these strategies were not analysed separately.

**Table 1.** General demographic and immunovirological characteristics of CoRISpe and CoRISpe-FARO patients at last clinical visit since 2020

Demographic characteristics	Patients with ELHIV (*N* = 642)
Sex (male)	296/642 (46.1)
Age, y	24 [17–30]
Young adults (≥18 y)	434/642 (67.6)
Adolescents (12–18 y)	161/642 (25.1)
Children (<12 y)	47/642 (7.3)
Born in Spain	493/642 (76.8)
Ethnicity (Caucasian)	409 (65.3)
Age at HIV diagnosis, mo	10 [3–38]
Route of HIV transmission (vertical)	601/642 (93.6)
Reported death	1/642 (0.15)
***ART experience***	
Age at ART start, y	1.93 [0.38–5.03]
Receiving ART	636/642 (99.1)
Time under ART, y	22 [14–26]
ART companion drug	
2 NRTIs + 1 NNRTI	54/642 (8.6)
2 NRTIs + 1 PI	92/642 (14.6)
2 NRTIs + 1 INSTI	366/642 (58)
Others	109/642 (17.6)
***Virological features***	
Suppressed (all patients)	519/642 (80.8)
VF (all patients)	69/642 (10.7)
Suppressed (patients on ART)	624/636 (81.1)
VF (patients on ART)	69/636 (10.5)
***Immunological features***	
CD4 nadir, cells/mm^3^	310 [115–468]
CD4, cells/mm^3^	771 [573–1004]
% CD4	35 [28–41]
CD8, cells/mm^3^	765 [585–1033]
% CD8	34 [28–42]
CD4/CD8 ratio	1.01 [0.72–1.4]
CD4/CD8 ratio <1	280/587 (47.7)
Immunological category	
1 (CD4 ≥ 500 cells/mm^3^)	512/637 (80.4)
2 (200 ≤ CD4 < 500 cells/mm^3^)	100/637 (15.7)
3 (CD4 < 200 cells/mm^3^)	25/637 (3.9)
***Coinfections***	
Previous HCV infection	44/106 (8.2)
HCV RNA detected	0/44 (0)
Previous HBV infection	5/106 (0.9)

Values are shown as median [IQR] for continuous variables or number (%) for categorical variables.

In the last available clinical report since 2020, among the patients on ART, 81.1% were virologically suppressed and 10.5% were in confirmed VF. Among patients on ART with a detectable VL at the last clinical visit, 8% (*n* = 51) did not have a confirmatory second VL measurement and were therefore not classified as VF. These cases may represent transient blips or early VF requiring further follow-up. The median CD4 nadir was 310 cells/mm^3^ (IQR: 115–468), and the medians of CD4 cells and CD8 cells were 771 (IQR: 573–1004) cells/mm^3^ and 765 (IQR: 585–1033) cells/mm^3^, respectively. The median CD4/CD8 ratio was 1.01 (IQR: 0.72–1.4), and 47.7% did not achieve immune recovery (CD4/CD8 ratio <1). The frequency of patients in each immunological category based on CD4 T cell count was 80.4% in category 1 (CD4 ≥ 500 cells/mm^3^), compared with 15.7% and 3.9% in categories 2 (200≤ CD4 < 500 cells/mm^3^) and 3 (CD4 < 200 cells/mm^3^), respectively.

Among patients with available data (*n* = 106), 8.2% had a documented previous coinfection with HCV but none of them had a current active infection (negative HCV RNA) due to spontaneous clearance, successful IFN-based regimens or, more recently, thanks to new direct antiviral agents as we previously described.^27–29^ The prevalence of previous HBV infection was 0.9%. Information on HBV vaccination status was not available.

**Figure 1.**
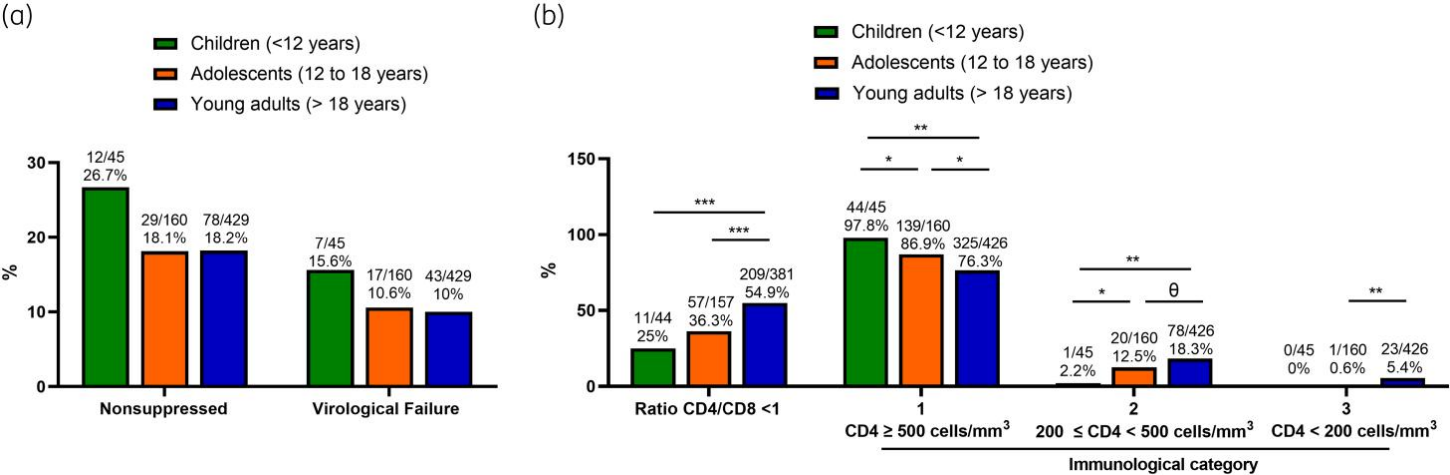
Immunovirological status by age groups of patients on ART for at least 6 months. Virological status including (a) confirmed virological failure and (b) immunological status based on CD4/CD8 ratio and immunological categories by age groups. Only individuals on ART for at least 6 months were included. Virological suppression was defined as HIV-1 RNA ≤50 copies/mL at the last available clinical visit. ‘Nonsuppressed’ refers to individuals with a detectable viral load (>50 copies/mL) at the last visit, including both patients with confirmed virological failure (defined as two consecutive viral load measurements >50 copies/mL) and individuals with a single detectable viral load without confirmatory testing, who were not classified as virological failure according to the predefined study criteria. Differences between age categories were determined using the chi-square test. Only comparisons with *P* values <0.1 are indicated. θ = 0.1 > *P* value ≥ 0.05; *0.01 ≤ *P* value < 0.05; **0.001 ≤ *P* value < 0.01; ***0.0001 ≤ *P* value < 0.001.

To further analyse differences in immunovirological status based on the patients’ age at the time of their last clinical visit, participants were classified into children (<12 years), adolescents (12–18 years) and young adults (>18 years), and only those on ART for at least 6 months were included in the analysis. Detailed data about immunovirological status based on age categories and group comparisons can be found in Table S1 (available as Supplementary data at *JAC* Online). Although there were no significant differences between groups, the prevalence of detectable VL (26.7%) and confirmed VF (15.6%) was higher in children compared with adolescents and young adults (Figure 1a).

Regarding immunological status, children exhibited enhanced immune recovery compared with adolescents and young adults, with adolescents also demonstrating a better profile than young adults (Figure 1b). Specifically, children and adolescents had a lower frequency of a CD4/CD8 ratio <1 compared with young adults. In terms of immunological categories, the frequency of individuals in category 1 was significantly higher for children (97.8%) than for adolescents (86.9%) and young adults (76.3%) (*P* < 0.001 for both comparisons). No children were in category 3, and the frequency of adolescents in this category (0.6%) was significantly lower than that observed for young adults (5.4%) (*P* = 0.009).

### Factors associated with VF and immunological status

**Figure 2.**
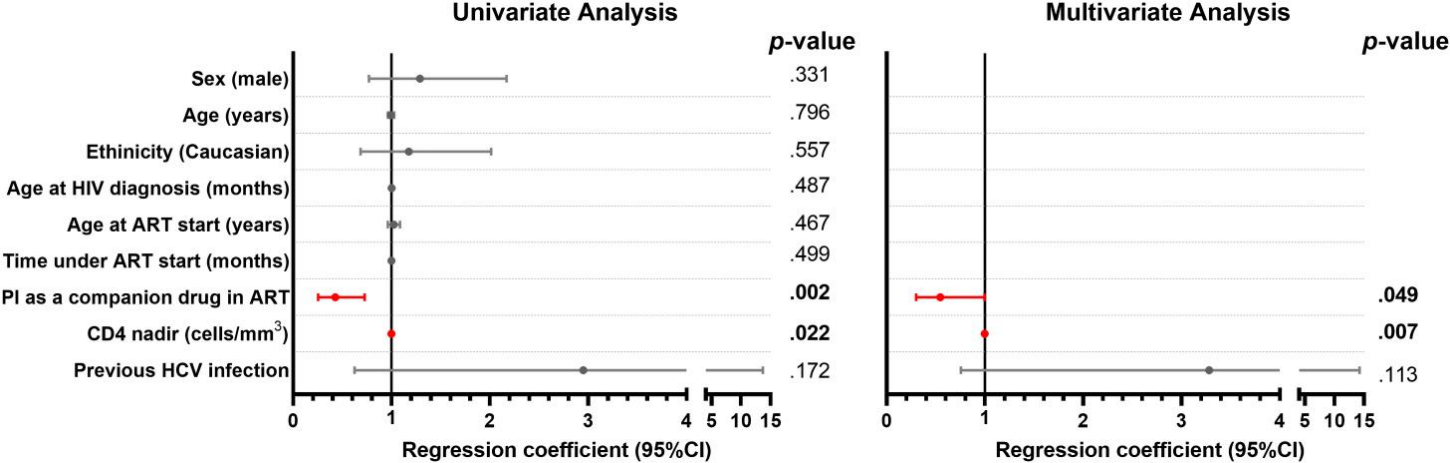
Factors associated with virological failure at the last clinical visit. Individuals on ART for at least 6 months were selected; participants with detectable viral load at the last clinical visit but with no confirmed VF (two consecutive viral load determinations above detection limit) were excluded from the analysis (virologically suppressed, *n* = 515; confirmed VF, *n* = 67). Virological failure was defined as two consecutive viral loads >50 copies/mL. Data on forest plots were calculated by univariate (left) and multivariate (right) logistic regression models after adjustment for all variables with a *P* value <0.2 in the univariate analysis. Abbreviations: ART, antiretroviral treatment; PI, protease inhibitors; 95% CI, 95% of confidence interval; *P*-value, level of significance. Dots indicate the point estimates of the regression coefficients, and horizontal lines represent the 95% CI. Variables highlighted in red correspond to factors that remained statistically significant (*P*<0.05).

Factors associated with VF in ELHIV individuals are displayed in Table S2. Only ELHIV individuals on ART for at least 6 months were selected; participants with detectable VL at the last clinical visit but with no confirmed VF (two consecutive VL determinations above detection limit) were excluded from the analysis (ELHIV virologically suppressed, *n* = 515; and ELHIV with confirmed VF, *n* = 67). A regimen including a PI and lower nadir CD4 were associated with VF, whereas a previous HCV infection was protective against VF. After adjustment for all variables with a *P* value <0.2 in univariate analysis, only the use of a PI as a companion drug in ART and a lower nadir CD4 remained independently associated with VF [adjusted regression coefficient 0.545 (95% CI, 0.299–0.995), *P* = 0.049; and 0.998 (95% CI, 0.997–0.999), *P* = 0.007; respectively] (Figure 2). PI-based regimens mainly included boosted darunavir, atazanavir and lopinavir, reflecting their use in patients with extensive treatment histories. The same analysis segregating the three groups according to age (children, adolescents and young adults) showed the same associations separately except in the case of the adolescent group, where we found an association between the use of PI and VF but not with low nadir (Table S3).

**Figure 3.**
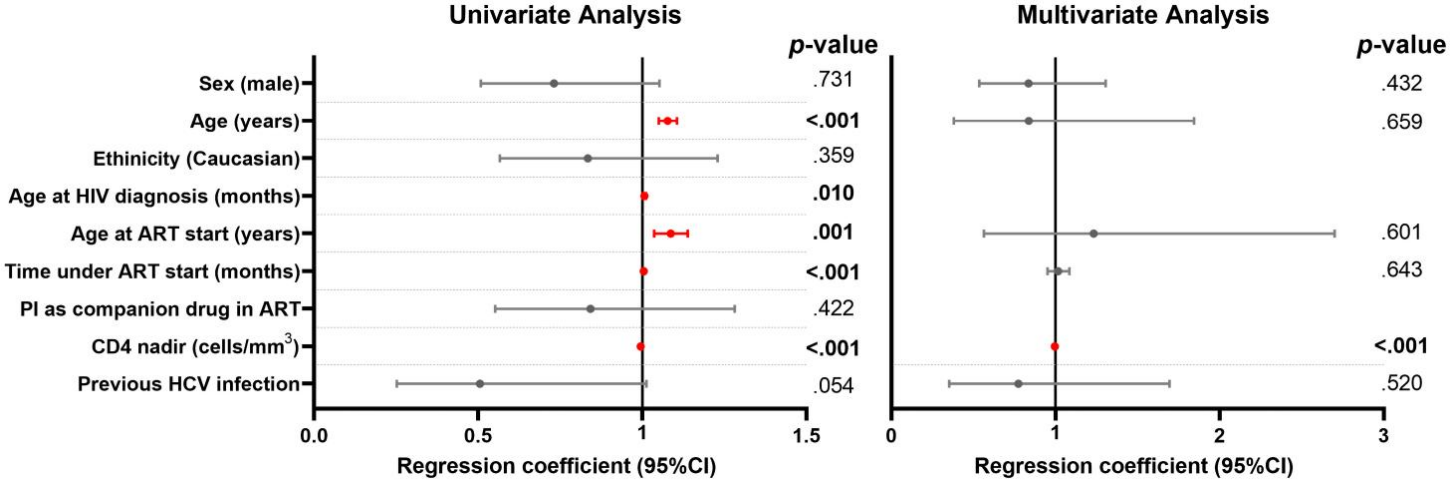
Factors associated with a CD4/CD8 ratio <1 at the last clinical visit. Individuals on ART for at least 6 months, virologically suppressed and with available data for CD4/CD8 ratio were included in the analysis (individuals with CD4/CD8 ratio >1, *n* = 268; with CD4/CD8 ratio <1, *n* = 208). Data for forest plots were calculated by univariate (left) and multivariate (right) logistic regression models after adjustment for all variables with a *P* value <0.2 in the univariate analysis. Since age at ART start and age at HIV diagnosis are strongly correlated, only age at ART start was included in the multivariate model when appropriate. ORs were not calculated when no cases were found in some variables. Abbreviations: ART, antiretroviral treatment; PI, protease inhibitors; 95% CI, 95% of confidence interval; *P*-value, level of significance. Dots indicate the point estimates of the regression coefficients, and horizontal lines represent the 95% CI. Variables highlighted in red correspond to factors that remained statistically significant (*P*<0.05).

For the analysis of factors associated with impaired immune recovery (CD4/CD8 ratio <1) in the general population, only individuals on ART for at least 6 months, who were virologically suppressed and with available data were selected (ELHIV with CD4/CD8 ratio >1, *n* = 268; ELHIV with CD4/CD8 ratio <1, *n* = 208) (Table S4). Older age at the last visit, at HIV diagnosis and at ART start, as well as longer time under ART and lower nadir CD4 were associated with impaired immune recovery. After adjustment for all variables with a *P* value <0.2 in univariate analysis, only lower nadir CD4 remained independently associated [0.996 (95% CI, 0.994–0.997), *P* < 0.001] (Figure 3). Since age at ART start and age at HIV diagnosis are strongly correlated, only age at ART start was included in the multivariate model. In this case, the separate analysis based on age groups showed marked differences in the associated factors. Although no variable was associated with immune recovery in children, probably due to the low number of subjects with impaired immune recovery and available data, in adolescents non-Caucasian ethnicity, older age at ART start and low nadir CD4 were independently associated with impaired immune recovery [3.705 (95% CI, 1.416–9.69), *P* = 0.008; 1.468 (95% CI, 1.101–1.956), *P* = 0.009; and 0.996 (95% CI, 0.993–1.040), *P* = 0.001; respectively]; and in young adults only low nadir CD4 remained independently associated with impaired immune recovery in the adjusted multivariate analysis recovery [0.996 (95% CI, 0.994–0.997), *P* < 0.001]. Detailed data can be found in Table S5.

**Figure 4.**
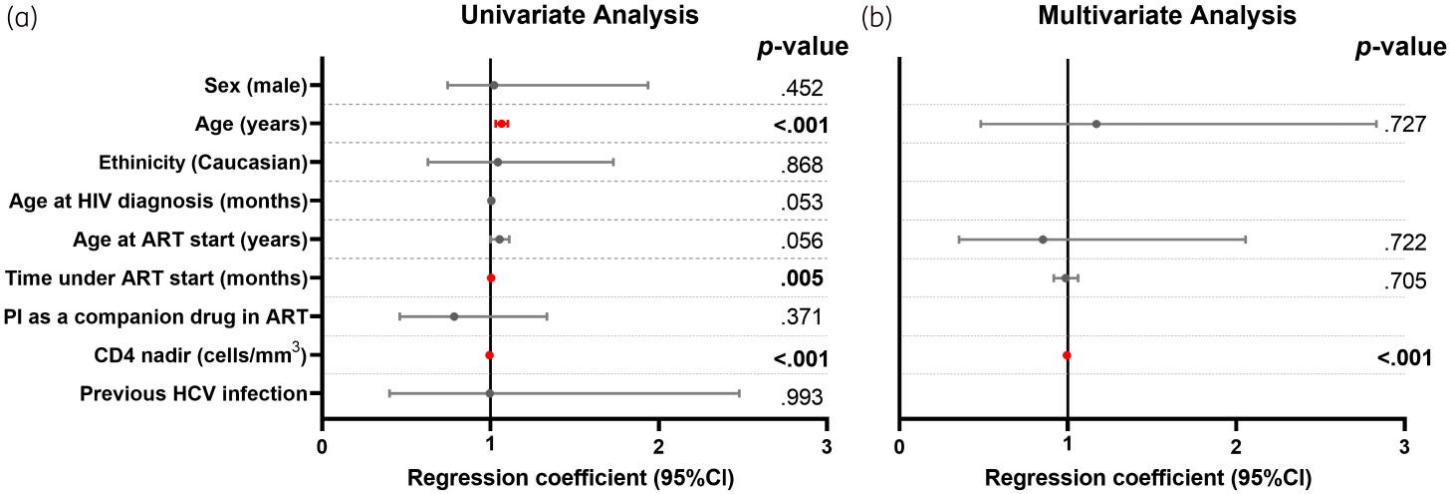
Factors associated with immunological category at the last clinical visit. Individuals on ART for at least 6 months and virologically suppressed were included in the analysis (individuals in immunological category 1, CD4 ≥ 500 cells/mm^3^, *n* = 430; individuals in immunological categories 2 and 3, CD4 < 500 cells/mm^3^, *n* = 82). Data on forest plots were calculated by the univariate (left) and multivariate (right) logistic regression model after adjustment by all variables with *P*-value <0.2 in univariate analysis. Since age at ART start as well as age at HIV diagnosis strongly correlated, only age at ART start was included in the multivariate model when appropriate. Abbreviations: ART, antiretroviral treatment; PI, protease inhibitors; 95% CI, 95% of confidence interval; *P*-value, level of significance. Variables highlighted in red correspond to factors that remained statistically significant (*P*<0.05).

When analysing factors associated with immunological category based on CDC criteria, only individuals on ART for at least 6 months and virologically suppressed were selected and classified into two groups: (i) being in category 1 (*n* = 430) or (ii) being in category 2 or 3 (*n* = 82). Older age at the last clinical visit, longer time under ART and lower nadir CD4 were significantly associated with immunological categories 2 and 3, whereas older age at HIV diagnosis and ART start remained associated but the *P* values were close to borderline significance (*P* = 0.053 and *P* = 0.056, respectively). After adjustment for all variables with a *P* value <0.2 in univariate analysis, only the lower nadir CD4 remained independently associated with immunological categories 2 and 3 [0.994 (95% CI, 0.993–0.996), *P* < 0.001] (Figure 4; Table S6). Since age at ART start and age at HIV diagnosis are strongly correlated, only age at ART start was included in the multivariate model. Repeating the same analysis but segregating groups according to age, in adolescents and young adults we found the same association with a lower nadir CD4 in the adjusted analysis [0.996 (95% CI, 0.993–0.999), *P* = 0.01; and 0.994 (95% CI, 0.992–0.996), *P* < 0.001, respectively] (Table S7). Since there were no children in immunological categories 2 and 3 and meeting the previously defined selection criteria, the sub-analysis could not be performed on this group of children.

## Discussion

This study provides a comprehensive overview of the clinical, virological and immunological status of ELHIV in Spain since 2020. By analysing data from the CoRISpe and CoRISpe-FARO cohort, we identified key factors associated with VF and impaired immune recovery, offering valuable insights for optimizing care in this population.

### Virological outcomes

Our results indicate that virological suppression was achieved in 81.1% of patients receiving ART, with 10.5% experiencing confirmed VF. These findings align with previous studies highlighting the challenges of maintaining virological control in ELHIV individuals, particularly during childhood and adolescence,^7–9^ when adherence issues are more prevalent. Children showed the highest proportion of confirmed VF. This finding deserves consideration and should be interpreted in light of age-specific factors influencing virological control. In children, VF is often driven by factors distinct from those affecting adolescents and adults, including caregiver-dependent adherence, challenges related to drug formulations (such as palatability and dosing complexity), and the need for dose adjustments associated with rapid growth and weight changes.^7,30–32^ In addition, younger children are more likely to receive PI-based regimens, which, although effective, may be associated with higher pill burden, poorer tolerability and adherence challenges.^33–35^

Consistent with recent observational studies^36^ and meta-analyses,^37^ our study highlights the role of PI-based regimens as a risk factor for VF, whereas regimens containing INSTIs appear to be protective. The association between PI-based regimens and VF should be interpreted with caution. This age-independent association between PI use and poorer virological outcomes may be linked to increased treatment disruptions compared with other regimens, particularly among children and adolescents.^33,38^ Several factors contribute to this, including PIs’ poor tolerability and taste, regimen complexity (such as pill burden, storage requirements and dosing frequency)^30,31,34,35,39–41^ as well as the risk of drug–drug interactions and adverse effects, such as gastrointestinal symptoms.^42^ In addition, PI-based therapies are frequently prescribed to heavily pre-treated individuals, often with prior VF or drug resistance. Therefore, PI use is more likely to represent a marker of complex treatment history and patient characteristics rather than an independent causal risk factor for VF. On the contrary, INSTIs have demonstrated high potency in controlling viral replication, even in reservoir cells, and a high genetic barrier to resistance, while also being less toxic.^43^ This improved safety profile contributes to better adherence, ultimately leading to enhanced virological control in patients receiving INSTI-based regimens. Notably, in the absence of adherence and resistance data, the VF observed among young adults cannot be solely attributed to poor adherence but may also be related to their longer duration on ART compared with younger patients. This highlights the importance of regular VL monitoring, timely resistance testing, and adherence assessment in cases of VF. Clinicians should carefully consider drug pharmacology and adherence when prescribing ART regimens to optimize treatment outcomes. They must also tailor treatments based on the patient’s age and promptly identify adherence issues to prevent treatment failure. Notably, lower CD4 nadir values were also independently associated with VF, highlighting the significance of preserved immune status for effective viral suppression and early ART initiation to mitigate long-term immune depletion and comorbidities.^44–46^

### Immunological recovery

Immunological recovery, as assessed by the CD4/CD8 ratio, remained suboptimal in a substantial proportion of participants. Almost half (47.7%) of the cohort had a CD4/CD8 ratio <1, a marker in ELHIV associated with immune senescence, inflammation,^20,21,47^ and increased risk of non-AIDS-related complications.^45,48,49^ These findings align with previous reports showing that ELHIV individuals face persistent immune activation despite virological suppression.^20,50^ Research suggests that children who start ART at an early age tend to follow a CD4 trajectory similar to that of the general population,^51,52^ and higher CD4 counts at ART initiation have been linked to higher long-term CD4 levels^53^ and improved CD4 recovery after treatment interruptions.^54^ Consistent with these observations, our analysis confirms that a lower CD4 nadir is the stronger predictor of impaired immune recovery across all age groups, supporting the importance of early and effective ART initiation. Perinatally HIV cohorts represent a unique opportunity to examine sex-based differences due to the similar prevalence between males and females. Previous studies in children^55^ and adults^56^ have suggested that females exhibit a better immunovirological response than males; the modulatory effects of sex hormones on immune activation, differences in thymic output, and variations in inflammatory and senescence pathways are among proposed mechanisms. Although our study found a higher prevalence of males with a CD4/CD8 ratio <1, sex was not significantly associated with immune recovery in the multivariate analysis. This lack of association may be partly explained by limited statistical power to detect sex-related differences and by a cohort enriched with individuals showing good immunological status. Therefore, in this population, markers of disease severity and ART history, particularly a CD4 nadir, may play a more prominent role in long-term immune recovery than sex alone.

### Age-related differences

A stratified analysis revealed notable differences in virological and immunological outcomes across age groups. Children showed the highest rates of immune recovery, with 97.8% achieving CD4 counts ≥500 cells/mm³, compared with 86.9% of adolescents and 76.3% of young adults. Although enhanced thymic function and a more diverse T cell receptor repertoire in younger individuals may contribute to these differences,^57^ the present outcomes are more likely the result of a combination of factors, including earlier ART initiation. Importantly, in our multivariate analyses including all age groups, chronological age itself was not independently associated with immune recovery once markers of disease severity and ART history were taken into account. This finding supports the notion that cumulative immune damage, reflected by a CD4 nadir, and ART-related factors play a more prominent role in shaping long-term immunological outcomes than age alone.

Adolescents and young adults were disproportionately affected by VF and impaired immune recovery, a pattern consistent with findings from other European and global cohort studies.^5,52,58^ These studies indicate that young adults with ELHIV who initiated ART before the age of 10 and had a lower CD4 nadir in childhood experienced a decline in CD4 count over time. In contrast, those who started ART before age 10 with a higher nadir CD4 were more likely to achieve CD4 levels in adolescence and early adulthood comparable to those of their peers in the general population. This highlights the need for targeted interventions aimed at these vulnerable groups and suggests that, in children, optimizing ART in addition to early initiation may be crucial for maintaining and maximizing good immune function and reconstitution later in life. The challenges faced by adolescents are especially critical, as adherence difficulties during this stage significantly impact on long-term outcomes. Consistent with previous European studies, these results emphasize the importance of robust adherence support and structured transition programmes to assist this high-risk population.^8,9^

### Implications for care and global targets

The present analysis uses the UNAIDS 90-90-90 global targets for 2020 as a reference framework, as updated data were available from that year through December 2023. Had the more stringent 95-95-95 targets been applied,^59^ these gaps would likely appear even more pronounced, underscoring the challenges faced by this population in fully translating global HIV goals into clinical practice. The findings emphasize the critical importance of early ART initiation and sustained virological suppression to prevent long-term immune dysfunction in ELHIV individuals. Although this study was conducted in a high-income setting with universal ART access, the observed gaps in virological and immunological outcomes highlight areas requiring intervention even in resource-rich contexts. Clinicians should consider the potential trade-offs of different ART regimens at early ages, particularly the increased risk of VF associated with PI-based treatments, to optimize long-term immune reconstitution. Currently, new drug classes, such as INSTIs, now recommended as the preferred therapy in guidelines, may offer improved virological and immunological control in the paediatric population from the earliest years of life. Further research is needed to better understand the mechanisms linking PI use to poorer virological outcomes and to develop strategies that enhance treatment efficacy and durability. Additionally, strategies to improve adherence during adolescence, such as peer support, digital interventions, new formulations (e.g. long-acting injectables) and structured transition programmes, could play a pivotal role in improving outcomes.

### Limitations and future research

This study’s retrospective and cross-sectional design makes it difficult to establish causality, particularly between PI use and VF. The lack of adherence and resistance data also limits a deeper analysis of treatment failure mechanisms. The analysis was restricted to individuals alive and actively followed since 2020, introducing an inherent survivorship bias. This limitation is particularly relevant for the adult population, who represent a selected group of long-term survivors from earlier birth cohorts exposed to less effective and more toxic ART. Consequently, immunovirological outcomes in young adults may not be fully representative of the entire population of individuals with ELHIV.

Moreover, the study period (2020–2023) overlapped with the COVID-19 pandemic, which may have influenced patterns of clinical follow-up and laboratory monitoring. Disruptions in routine care, changes in visit frequency, and delays in virological assessments have been reported during this period and could have affected the availability of confirmatory VL measurements. As a result, a proportion of individuals with a single detectable VL could not be classified as VF according to the predefined criteria. Although this conservative approach may have led to a slight underestimation of VF, it reduces the risk of misclassifying transient viral blips as VF. In a paediatric and adolescent cohort in Madrid, clinical stability and good virological control were maintained during the pandemic period, particularly among older patients, potentially reflecting sustained or improved adherence to ART while remaining at home with family support.^60^ These findings support the notion that, although the pandemic may have affected follow-up logistics and data completeness, its impact on virological outcomes in this population was likely limited. Furthermore, the absence of comorbidity and biomarker data further restricts our understanding of immune dysfunction. Although the cohort includes individuals followed since childhood, the present analysis was not designed to explore cumulative treatment exposure or longitudinal patterns of VF. Future longitudinal studies are warranted to address these important questions, particularly in adults with ELHIV.

Lastly, as this study was conducted in a high-income setting, the findings may not be generalizable to resource-limited contexts. Future longitudinal and comparative studies are needed to validate these results globally.

### Conclusions

This analysis highlights the persistent challenges faced by ELHIV individuals in achieving optimal immunovirological outcomes. Early ART initiation, adherence support and regimen optimization, through an expanded therapeutic arsenal with drugs tailored for children and adolescents, remain essential strategies for improving long-term health outcomes. The insights gained from this study can inform targeted interventions to enhance care and quality of life for this unique population while contributing to global efforts toward UNAIDS targets.

## Supplementary Material

dkag079_Supplementary_Data

## Data Availability

The main datasets supporting the conclusions of this article are included within the article and its additional files. The datasets generated during and/or analysed during the current study are available from the corresponding author on reasonable request.

## Supplementary data

Tables S1 to S7 are available as Supplementary data at *JAC* Online.
