# Supplementary material for: Clinical and immunovirological status in children, adolescents and young adults with early-life-acquired HIV: a Spanish multi-cohort analysis since 2020
Source: J Antimicrob Chemother. 2026 Mar 11;81(4):dkag079. doi: 10.1093/jac/dkag079 (PMC13016914; doi:10.1093/jac/dkag079)
Supplement: dkag079_Supplementary_Data [file dkag079_supplementary_data.docx]

**Clinical and immunovirological status in children, adolescents and young adults with early-life acquired HIV: a Spanish multi-cohort analysis since 2020**

**Supplementary material.**

**Supplementary Table 1.** **Immunovirological status by age categories on patients on ART for at least 6 months.**

|  |  |  |  | ***p*-value** | | |
| --- | --- | --- | --- | --- | --- | --- |
|  | **Children (<12 years)**  **n=45** | **Adolescents (12 to 18 years) n=160** | **Young adults (> 18 years) n=429** | **Children vs Adolescents** | **Children vs Young adults** | **Adolescents vs Young adults** |
| **Nonsuppressed** | 12/45 (26.7) | 29/160 (18.1) | 78/429 (18.2) | .206 | .167 | .987 |
| **VF** | 7/45 (15.6) | 17/160 (10.6) | 43/429 (10) | .363 | .250 | .830 |
| **Ratio CD4/CD8 <1** | 11/44 (25) | 57/157 (36.3) | 209/381 (54.9) | .161 | **<.001** | **<.001** |
| **Immunological category** |  |  |  |  |  |  |
| **1 (****CD4 ≥ 500 cells/mm^3^)** | 44/45 (97.8) | 139/160 (86.9) | 325/426 (76.3) | **.037** | **.001** | **.005** |
| **2 (200 ≤ CD4 < 500 cells/mm^3^)** | 1/45 (2.2) | 20/160 (12.5) | 78/426 (18.3) | **.045** | **.006** | .093 |
| **3 (CD4 < 200 cells/mm^3^)** | 0/45 (0) | 1/160 (0.6) | 23/426 (5.4) | .595 | .110 | **.009** |

Analysis includes patients on ART for at least 6 months. Virological suppression was defined as HIV-1 RNA ≤50 copies/mL at the last available clinical visit. *Nonsuppressed* refers to individuals with a detectable viral load (>50 copies/mL) at the last visit, including both patients with confirmed virological failure (defined as two consecutive viral load measurements >50 copies/mL) and individuals with a single detectable viral load without confirmatory testing, who were not classified as virological failure according to the predefined study criteria. Immunological category: 1) CD4 ≥ 500 cells/mm^3^), 2) (200 ≤ CD4 < 500 cells/mm^3^), and 3) (CD4 < 200 cells/mm^3^). Variables are expressed as numbers and percentages. Abbreviations: VF, virological failure. Chi-square test was used for comparisons. *p*-value < 0.05 are highlighted in bold.

**Supplementary Table 2. Factors associated with VF at last clinical visit.**

|  |  |  | **Univariate Analysis** | **Multivariate Analysis** |
| --- | --- | --- | --- | --- |
|  | **Virologically suppressed (n=515)** | **Confirmed VF (n=67)** | **p; OR (95% CI)** | **p; aOR (95% CI)** |
| **Sex (male)** | 240 (46.6) | 27 (40.3) | 0.331; 1.29 (0.77 to 2.17) |  |
| **Age (years)** | 24 [17-29] | 24 [16-30] | 0.796; 0.99 (0.964 to 1.028) |  |
| **Ethnicity (Caucasian)** | 333 (66.2) | 40 (62.5) | 0.557; 1.175 (0.686 to 2.014) |  |
| **Age at HIV diagnosis (months)** | 9.5 [3-34] | 15 [3-51] | 0.487; 1.002 (0.995 to 1.008) |  |
| **Age at ART start (years)** | 1.8 [0.38-4.7] | 2.85 [0.35-5.8] | 0.467; 1.023 (0.963 to 1.086) |  |
| **Time under ART (months)** | 271 [178-316] | 278 [165-316] | 0.499; 0.999 (0.996 to 1.002) |  |
| **PI as a companion drug in ART** | 123 (23.9) | 28 (42.4) | **0.002****;** **0.429 (****0.253 to** **0.728)** | **0.049;** **0.545 (****0.299 to** **0.995)** |
| **CD4 nadir (cells/mm^3^)** | 317 [120-468] | 176 [50-403] | **0.022;** **0.999 (****0.997 to** **0.999)** | **0.007;** **0.998 (****0.997 to** **0.999)** |
| **Previous HCV infection** | 39 (7.6) | 2 (3) | 0.172; 2.95 (0.624 to 13.714) | 0.113; 3.281 (0.756 to 14.247) |

Only individuals on ART for at least 6 months were selected and participants with detectable viral load in the last clinical visit but with no confirmed VF (two consecutive viral load determinations above detection limit) were excluded from the analysis. VF was defined as 2 consecutive viral loads >50 copies/mL. Values are shown as median [IQR] for continuous variables or number (%) for categorical variables. Data calculated by univariate and multivariate logistic regression model after adjustment by all variables with p-value <0.2 in univariate analysis. Odds ratios were not calculated when no cases were found in some variables. Abbreviations: VF, virological failure; ART, antiretroviral treatment; PI, protease inhibitors; HCV, Hepatitis C Virus; 95% CI, 95% of confidence interval; p-value, level of significance. p-value < 0.05 are highlighted in bold.

**Supplementary table 3. Factors associated with VF in the last clinical visit per age group.**

|  | **Children (<12 years)** | | | |
| --- | --- | --- | --- | --- |
|  | **Virologically suppressed (n=34)** | **Confirmed VF (n=7)** | **Univariate Analysis** | **Multivariate Analysis** |
|  |  |  | **p; OR (95% CI)** | **p; aOR (95% CI)** |
| **Sex (male)** | 13 (38.2) | 3 (42.9) | 0.748; 0.762 (0.145 to 3.993) |  |
| **Ethnicity (Caucasian)** | 8 (24.2) | 3 (42.9) | 0.349; 0.444 (0.081 to 2.426) |  |
| **Age at HIV diagnosis (months)** | 2 [0-16] | 5.5 [0.75-55.5] | 0.230; 1.023 (0.986 to 1.061) |  |
| **Age at ART start (years)** | 0.3 [0.2-1.51] | 0.48 [0.06-4.32] | 0.304; 1.25 (0.817 to 1.91) |  |
| **Time under ART (months)** | 77 [54-116] | 64 [33-128] | 0.445; 0.99 (0.966 to 1.015) |  |
| **PI as a companion drug in ART** | 4 (12.1) | 3 (42.9) | 0.069; 0.184 (0.030 to 1.142) | **0.032; 0.065 (0.005 to 0.791)** |
| **CD4 nadir (cells/mm^3^)** | 677 [525-859] | 322 [203-679] | 0.06; 0.996 (0.992 to 1) | **0.036; 0.994 (0.989 to 1)** |
| **Previous HCV infection** | 0 (0) | 0 (0) |  |  |
|  | **Adolescents (12 to 18 years)** | | | |
|  | **Virologically suppressed (n=131)** | **Confirmed VF (n=17)** | **Univariate Analysis** | **Multivariate Analysis** |
|  |  |  | **p; OR (95% CI)** | **p; OR (95% CI)** |
| **Sex (male)** | 71 (53.8) | 8 (47.1) | 0.580; 1.331 (0.484 to 3.664) |  |
| **Ethnicity (Caucasian)** | 57 (43.5) | 3 (17.6) | **0.05; 3.644 (0.999 to 13.293)** | 0.063; 3.629 (0.933 to 14.12) |
| **Age at HIV diagnosis (months)** | 9 [2-34.2] | 18 [1-65.5] | 0.382; 1.005 (0.994 to 1.016) |  |
| **Age at ART start (years)** | 0.73 [0.17-3.31] | 2.56 [0.33-8.54] | 0.131; 1.092 (0.974 to 1.224) | 0.577; 0.918 (0.679 to 1.241) |
| **Time under ART (months)** | 176 [141-198] | 166 [82-182] | 0.101; 0.992 (0.984 to 1.004) | 0.3; 0.988 (0.966 to 1.011) |
| **PI as a companion drug in ART** | 16 (12.2) | 5 (29.4) | 0.065; 0.334 (0.104 to 1.073) | **0.038; 0.251 (0.068 to 0.923)** |
| **CD4 nadir (cells/mm^3^)** | 437 [311-599] | 403 [200-486] | 0.186; 0.998 (0.996 to 1.001) | 0.529; 0.999 (0.996 to 1.002) |
| **Previous HCV infection** | 1 (1.1) | 0 (0) | 0.580; 1.331 (0.484 to 3.664) |  |
|  | **Young Adults (>18 years)** | | | |
|  | **Virologically suppressed (n=351)** | **Confirmed VF (n=43)** | **Univariate Analysis** | **Multivariate Analysis** |
|  |  |  | **p; OR (95% CI)** | **p; OR (95% CI)** |
| **Sex (male)** | 158 (44.8) | 16 (37.2) | 0.35; 1.366 (0.711 to 2.624) |  |
| **Ethnicity (Caucasian)** | 269 (78.4) | 35 (87.5) | 0.348; 0.648 (0.262 to 1.603) |  |
| **Age at HIV diagnosis (months)** | 10.5 [3-36] | 10.5 [3-41] | 0.871; 1.001 (0.993 to 1.008) |  |
| **Age at ART start (years)** | 2.46 [0.63-5.34] | 2.97 [0.49-5.7] | 0.948; 1.003 (0.929 to 1.082) |  |
| **Time under ART (months)** | 299 [266-332] | 306 [282-347] | 0.341; 1.002 (0.997 to 1.008) |  |
| **PI as a companion drug in ART** | 103 (29.3) | 20 (47.6) | **0.02; 0.462 (0.242 to 0.884)** | **0.026; 0.478 (0.249 to 0.917)** |
| **CD4 nadir (cells/mm^3^)** | 220 [63-375] | 112 [28-251] | **0.021; 0.998 (0.996 to 1)** | **0.035; 0.998 (0.996 to 1)** |
| **Previous HCV infection** | 38 (12.1) | 2 (5.1) | 0.209; 2.559 (0.592 to 11.038) |  |

Only individuals on ART for at least 6 months were selected and participants with detectable viral load in the last clinical visit but with no confirmed VF (two consecutive viral load determinations above detection limit) were excluded from the analysis. VF was defined as 2 consecutive viral loads >50 copies/mL. Values are shown as median [IQR] for continuous variables or number (%) for categorical variables. Data calculated by univariate and multivariate logistic regression model after adjustment by all variables with p-value <0.2 in univariate analysis. Since age at ART start as well as age at HIV diagnosis strongly correlated, only age at ART start was included in the multivariate model when appropriate. Odds ratios were not calculated when no cases were found in some variables. Abbreviations: VF, virological failure; ART, antiretroviral treatment; PI, protease inhibitors; HCV, Hepatitis C Virus; 95% CI, 95% of confidence interval; p-value, level of significance. p-value < 0.05 are highlighted in bold.

**Supplementary Table 4. Factors associated with ratio CD4/CD8 < 1 at last clinical visit.**

|  |  |  | **Univariate Analysis** | **Multivariate Analysis** |
| --- | --- | --- | --- | --- |
|  | **CD4/CD8 ratio ≥ 1 (n=268)** | **CD4/CD8 ratio < 1 (n=208)** | **p; OR (95% CI)** | **p; aOR (95% CI)** |
| **Sex (male)** | 117 (43.7) | 107 (51.4) | 0.092; 0.731 (0.508 to 1.052) | 0.432; 0.836 (0.535 to 1.306) |
| **Age (years)** | 21 [15-28] | 27 [19-31] | **<0.001;** **1.077 (****1.050 to** **1.105)** | 0.659; 0.837 (0.380 to 1.844) |
| **Ethnicity (Caucasian)** | 167 (63.7) | 137 (68) | 0.359; 0.834 (0.566 to 1.229) |  |
| **Age at HIV diagnosis (months)** | 6 [2-25] | 14.5 [4-48] | **0.010;** **1.006 (****1.001 to** **1.011)** |  |
| **Age at ART start (years)** | 1.2 [0.25-3.6] | 2.4 [0.56-6.49] | **0.001;** **1.086 (****1.036 to** **1.138)** | 0.601; 1.233 (0.563 to 2.701) |
| **Time under ART (months)** | 231 [153-300] | 286 [203-324] | **<0.001;** **1.004 (****1.002 to** **1.006)** | 0.643; 1.016 (0.951 to 1.085) |
| **PI as a companion drug in ART** | 62 (23.1) | 54 (26.3) | 0.422; 0.842 (0.552 to 1.282) |  |
| **CD4 nadir (cells/mm^3^)** | 401 [260-599] | 191 [49-348] | **<0.001;** **0.995 (****0.994 to** **0.996)** | **<0.001;** **0.996 (****0.994 to** **0.997)** |
| **Previous HCV infection** | 15 (6.6) | 21 (12.3) | 0.054; 0.505 (0.252 to 1.012) | 0.520; 0.773 (0.352 to 1.695) |

Individuals on ART for at least 6 months, virologically suppressed and with available data for CD4/CD8 ratio were included in the analysis. Values are shown as median [IQR] for continuous variables or number (%) for categorical variables. Data calculated by univariate and multivariate logistic regression model after adjustment by all variables with p-value <0.2 in univariate analysis. Since age at ART start as well as age at HIV diagnosis strongly correlated, only age at ART start was included in the multivariate model when appropriate. Abbreviations: ART, antiretroviral treatment; PI, protease inhibitors; HCV, Hepatitis C Virus; 95% CI, 95% of confidence interval; p-value, level of significance. p-value < 0.05 are highlighted in bold.

**Supplementary table 5. Factors associated with CD4/CD8 ratio < 1 per age group in the last clinical visit.**

|  | **Children (<12 years)** | | | |
| --- | --- | --- | --- | --- |
|  | **CD4/CD8 ratio ≥ 1 (n=28)** | **CD4/CD8 ratio < 1 (n=4)** | **Univariate Analysis** | **Multivariate Analysis** |
|  |  |  | **p; OR (95% CI)** | **p; aOR (95% CI)** |
| **Sex (male)** | 9 (32.1) | 3 (75) | 0.131; 0.158 (0.014 to 1.737) |  |
| **Ethnicity (Caucasian)** | 7 (25.9) | 1 (25) | 0.968; 1.05 (0.093 to 11.824) |  |
| **Age at HIV diagnosis (months)** | 2 [0-14.2] | 15 [8-41] | 0.301; 1.026 (0.977 to 1.079) |  |
| **Age at ART start (years)** | 0.21 [0.01-1.25] | 1.03 [0.17-2.97] | 0.479; 1.23 (0.695 to 2.174) |  |
| **Time under ART (months)** | 74.5 [54-115] | 97.5 [74.5-129] | 0.344; 1.017 (0.983 to 1.052) |  |
| **PI as a companion drug in ART** | 3 (10.7) | 1 (25) | 0.434; 0.360 (0.028 to 4.659) |  |
| **CD4 nadir (cells/mm^3^)** | 689 [532-891] | 556 [396-624] | 0.201; 0.996 (0.991 to 1.002) |  |
| **Previous HCV infection** | 0 (0) | 0 (0) |  |  |
|  | **Adolescents (12 to 18 years)** | | | |
|  | **CD4/CD8 ratio≥1 (n=87)** | **CD4/CD8 ratio < 1 (n=42)** | **Univariate Analysis** | **Multivariate Analysis** |
|  |  |  | **p; OR (95% CI)** | **p; aOR (95% CI)** |
| **Sex (male)** | 43 (49.4) | 27 (64.3) | 0.114; 0.543 (0.254 to 1.159) | 0.743; 0.858 (0.342 to 2.151) |
| **Ethnicity (Caucasian)** | **47 (54.7)** | 10 (23.8) | **0.001; 3.856 (1.686 to 8.820)** | **0.008; 3.705 (1.416 to 9.69)** |
| **Age at HIV diagnosis (months)** | 5 [1-23] | 29 [9-112] | **<0.001; 1.021 (1.01 to 1.032)** |  |
| **Age at ART start (years)** | 0.53 [0.14-2.3] | 2.46 [0.38-9.7] | **<0.001; 1.223 (1.102 to 1.358)** | **0.009; 1.468 (1.101 to 1.956)** |
| **Time under ART (months)** | 176 [147-202] | 169 [82-197] | **0.016; 0.991 (0.984 to 0.998)** | 0.074; 1.019 (0.998 to 1.040) |
| **PI as a companion drug in ART** | 11 (12.6) | 5 (11.9) | 0.905; 1.071 (0.347 to 3.308) |  |
| **CD4 nadir (cells/mm^3^)** | 540 [349-673] | 343 [205-434] | **<0.001; 0.995 (0.993 to 0.997)** | **0.001; 0.996 (0.993 to 1.040)** |
| **Previous HCV infection** | 1 (1.5) | 0 (0) |  |  |
|  | **Young adults (>18 years)** | | | |
|  | **CD4/CD8 ratio ≥ 1 (n=153)** | **CD4/CD8 ratio < 1 (n=162)** | **Univariate Analysis** | **Multivariate Analysis** |
|  |  |  | **p; OR (95% CI)** | **p; aOR (95% CI)** |
| **Sex (male)** | 65 (42.5) | 77 (47.5) | 0.368; 0.815 (0.523 to 1.272) |  |
| **Ethnicity (Caucasian)** | 113 (75.8) | 126 (80.8) | 0.297; 0.747 (0.432 to 1.292) |  |
| **Age at HIV diagnosis (months)** | 8 [3-31] | 12 [3-37] | 0.948; 1 (0.994 to 1.005) |  |
| **Age at ART start (years)** | 2.2 [0.62-5.06] | 2.5 [0.56-5.59] | 0.455; 1.020 (0.968 to 1.076) |  |
| **Time under ART (months)** | 295 [263-322] | 302 [269-336] | 0.166; 1.002 (0.999 to 1.006) | 0.863; 1 (0.996 to 1.003) |
| **PI as a companion drug in ART** | 48 (31.4) | 48 (30.2) | 0.821; 1.057 (0.654 to 1.71) |  |
| **CD4 nadir (cells/mm^3^)** | 331 [175-444] | 151 [38-293] | **<0.001; 0.996 (0.994 to 0.997)** | **<0.001; 0.996 (0.994 to 0.997)** |
| **Previous HCV infection** | 14 (10.2) | 21 (14.7) | 0.261; 1.512 (0.735 to 3.11) |  |

Individuals on ART for at least 6 months, virologically suppressed and with available data for CD4/CD8 ratio were included in the analysis. Values are shown as median [IQR] for continuous variables or number (%) for categorical variables. Data calculated by univariate and multivariate logistic regression model after adjustment by all variables with p-value <0.2 in univariate analysis. Since age at ART start as well as age at HIV diagnosis strongly correlated, only age at ART start was included in the multivariate model when appropriate. Odds ratios were not calculated when no cases were found in some variables. Abbreviations: ART, antiretroviral treatment; PI, protease inhibitors; HCV, Hepatitis C Virus; 95% CI, 95% of confidence interval; *p*-value, level of significance. *p*-value < 0.05 are highlighted in bold.

**Supplementary Table 6. Factors associated with immunological category at last clinical visit.**

|  |  |  | Univariate Analysis | Multivariate Analysis |
| --- | --- | --- | --- | --- |
|  | **Immunological category 1 (n=430)** | **Immunological category 2 and 3 (n=82)** | **p; OR (95% CI)** | **p; aOR (95% CI)** |
| Sex (male) | 203 (47.2) | 35 (42.7) | 0.452; 1.021 (0.745 to 1.935) |  |
| Age (years) | 24 [17-29] | 27.5 [20-32] | **<0.001;** **1.066 (****1.031 to** **1.103)** | 0.727; 1.17 (0.483 to 2.833) |
| Ethnicity (Caucasian) | 280 (66.4) | 51 (65.4) | 0.868; 1.044 (0.628 to 1.73) |  |
| Age at HIV diagnosis (months) | 8 [2-32] | 17.5 [5-57] | 0.053; 1.005 (1 to 1.01) |  |
| Age at ART start (years) | 1.54 [0.37-4.3] | 2.67 [0.46-6.86] | 0.056; 1.053 (0.999 to 1.111) | 0.722; 0.852 (0.353 to 2.056) |
| Time under ART (months) | 263 [167-315] | 292 [224-320] | **0.005;** **1.004 (****1.001 to** **1.007)** | 0.705; 0.986 (0.916 to 1.061) |
| PI as a companion drug in ART | 100 (23.4) | 23 (28) | 0.371; 0.784 (0.461 to 1.335) |  |
| CD4 nadir (cells/mm^3^) | 339 [180-519] | 124 [31-239] | **<0.001;** **0.995 (****0.993 to** **0.996)** | **<0.001;** **0.994 (****0.993 to** **0.996)** |
| Previous HCV infection | 32 (8.8) | 6 (8.8) | 0.993; 0.996 (0.4 to 2.48) |  |

Individuals on ART for at least 6 months, virologically suppressed were included in the analysis. Values are shown as median [IQR] for continuous variables or number (%) for categorical variables. Data calculated by univariate and multivariate logistic regression model after adjustment by all variables with p-value <0.2 in univariate analysis. Since age at ART start as well as age at HIV diagnosis strongly correlated, only age at ART start was included in the multivariate model when appropriate. Odds ratios were not calculated when no cases were found in some variables. Abbreviations: ART, antiretroviral treatment; PI, protease inhibitors; HCV, Hepatitis C Virus; 95% CI, 95% of confidence interval; p-value, level of significance. p-value < 0.05 are highlighted in bold.

**Supplementary table 7. Factors associated with immunological category per age group in the last clinical visit.**

|  | **Adolescents (12 to 18 years)** | | | |
| --- | --- | --- | --- | --- |
|  |  |  | **Univariate Analysis** | **Multivariate Analysis** |
|  | **Immunological category 1 (n=117)** | **Immunological category 2 and 3 (n=14)** | **p; OR (95% CI)** | **p; aOR (95% CI)** |
| **Sex (male)** | 62 (53) | 9 (64.3) | 0.426; 0.626 (0.198 to 1.982) |  |
| **Ethnicity (Caucasian)** | 53 (45.7) | 4 (28.6) | 0.231; 2.103 (0.624 to 7.093) |  |
| **Age at HIV diagnosis (months)** | 8 [2-34] | 23.5 [2.5-114] | **0.047; 1.011 (1 to 1.022)** | 0.327; 1.006 (0.994 to 1.018) |
| **Age at ART start (years)** | 0.72 [0.18-3.3] | 1.3 [0.13-7.4] | 0.338; 1.067 (0.935 to 1.218) |  |
| **Time under ART (months)** | 175 [141-197] | 182 [86-204] | 0.646; 0.997 (0.987 to 1.008) |  |
| **PI as a companion drug in ART** | 13 (11.1) | 3 (21.4) | 0.275; 0.458 (0.113 to 1.860) |  |
| **CD4 nadir (cells/mm^3^)** | 460 [330-628] | 258 [181-398] | **0.003; 0.995 (0.992 to 0.998)** | **0.010; 0.996 (0.993 to 0.999)** |
| **Previous HCV infection** | 1 (1.2) | 1 (7.1) |  |  |
|  | **Young adults (>18 years)** | | | |
|  |  |  | **Univariate Analysis** | **Multivariate Analysis** |
|  | **Immunological category 1 (n=281)** | **Immunological category 2 and 3 (n=71)** | **p; OR (95% CI)** | **p; aOR (95% CI)** |
| **Sex (male)** | 129 (45.9) | 27 (38) | 0.234; 1.383 (0.811 to 2.358) |  |
| **Ethnicity (Caucasian)** | 219 (79.9) | 48 (72.7) | 0.203; 1.493 (0.806 to 2.768) |  |
| **Age at HIV diagnosis (months)** | 9 [3-36] | 18 [5-55] | 0.348; 1.003 (0.997 to 1.009) |  |
| **Age at ART start (years)** | 2.2 [0.63-4.9] | 3.4 [0.7-6.8] | 0.310; 1.031 (0.972 to 1.094) |  |
| **Time under ART (months)** | 299 [269-332] | 307 [268-341] | 0.403; 1.002 (0.998 to 1.006) |  |
| **PI as a companion drug in ART** | 83 (30) | 20 (29.4) | 0.929; 1.027 (0.574 to 1.837) |  |
| **CD4 nadir (cells/mm^3^)** | 279 [95-421] | 71 [22-207] | **<0.001; 0.994 (0.992 to 0.996)** | **<0.001; 0.994 (0.992 to 0.996)** |
| **Previous HCV infection** | 31 (12.1) | 6 (10.3) | 0.707; 1.194 (0.474 to 3.010) |  |

Individuals on ART for at least 6 months, virologically suppressed were included in the analysis. Values are shown as median [IQR] for continuous variables or number (%) for categorical variables. Data calculated by univariate and multivariate logistic regression model after adjustment by all variables with p-value <0.2 in univariate analysis. Since age at ART start as well as age at HIV diagnosis strongly correlated, only age at ART start was included in the multivariate model when appropriate. Odds ratios were not calculated when no cases were found in some variables. Abbreviations: ART, antiretroviral treatment; PI, protease inhibitors; HCV, Hepatitis C Virus; 95% CI, 95% of confidence interval; p-value, level of significance. p-value < 0.05 are highlighted in bold.
